# Higher dietary choline and betaine intakes are associated with lower likelihood of central nervous system demyelination in Australian women

**DOI:** 10.1007/s00394-026-03915-x

**Published:** 2026-02-14

**Authors:** Paul Bailey, Hajar Mazahery, Lucinda J. Black, Alison Daly, Yasmine Probst, Ingrid van der Mei, Eleanor Dunlop

**Affiliations:** 1 Curtin School of Population Health, Curtin University, Perth, WA Australia; 2 Institute for Physical Activity and Nutrition (IPAN), School of Exercise and Nutrition Sciences, Deakin University, Geelong, VIC Australia; 3 enAble Institute, Curtin University, Perth, WA Australia; 4 Faculty of Science, Medicine and Health, University of Wollongong, Wollongong, NSW Australia; 5 Menzies Institute for Medical Research, University of Tasmania, Hobart, TAS Australia

**Keywords:** Choline, Betaine, Diet, Multiple sclerosis

## Abstract

**Purpose:**

Choline and betaine may be neuroprotective; however, the role of choline and betaine obtained from food in multiple sclerosis (MS) onset in humans is unclear. We aimed to test associations between intake of choline and betaine from food and likelihood of a first clinical diagnosis of central nervous system demyelination (FCD).

**Methods:**

We used data from the Ausimmune Study, a large, multi-centre case–control study conducted in four latitudinally diverse regions of Australia. Choline and betaine intakes were derived from food composition data sourced globally and dietary intake data collected using a self-administered, semiquantitative food frequency questionnaire. We used the alternate Mediterranean diet score (aMED) as a measure of diet quality. We applied logistic regression with full propensity score matching (264 cases, 474 controls) and stratified by sex (females: 205 cases/368 controls; males 59 cases/106 controls) to test associations between dietary choline and betaine intakes (per 50 mg/day) and likelihood of an FCD. Models were adjusted for age, region, education, smoking history, history of infectious mononucleosis, deseasonalised serum 25-hydroxyvitamin D concentration, total energy intake, dietary misreporting and aMED, and bootstrapped (500 replicates).

**Results:**

Independent of an overall healthy diet, higher choline intake (OR = 0.91; 95%CI 0.84–1.00; *p* = 0.05) and higher combined choline + betaine intake (OR = 0.92; 95%CI 0.86–0.99; *p* = 0.03) were significantly associated with lower likelihood of FCD in females only. We found no statistically significant associations in males (small sample).

**Conclusion:**

Given the potential beneficial role of choline and betaine in MS, investigation of associations and any diet- or sex-specific influences in other populations is warranted.

**Supplementary Information:**

The online version contains supplementary material available at 10.1007/s00394-026-03915-x.

## Introduction

Multiple sclerosis (MS) is an inflammatory immune-mediated disease that is characterised by demyelination of the central nervous system [1]. The resulting symptoms of MS are wide-ranging and include visual, sensory and mobility loss, limb weakness and fatigue [1]. Established risk factors for MS include a history of mononucleosis, or tobacco exposure, or obesity, or geographical latitude—possibly relating to sun exposure and/or vitamin D status [1]. There is increasing evidence to suggest that diet plays a role in the onset of MS. While dietary patterns that feature higher consumption of whole foods and lower consumption of nutrient-poor, highly processed and pro-inflammatory foods have been associated with lower likelihood of MS onset [2–6], it remains unclear which foods and food components drive such associations.

Choline is an essential nutrient that is naturally present in whole foods, particularly those of animal origin. It is most highly concentrated in meat (especially liver), egg yolk and fish, which are also the key foods that commonly contribute to choline intake at the population level [7, 8]. Smaller amounts of choline are found in some fruits and vegetables [7, 8]. Broadly, choline is important for brain, liver and muscle function [9]. Supplemental choline has been found to promote regeneration of demyelinated neurons in a study of animal models of MS [10]. In that study, toxic cuprizone-induced mouse models were treated with cytidine-S-diphosphate choline (CDP-choline). Doses of 50 and 100 mg/kg CDP choline—equivalent to doses deemed safe for human clinical trials—were found to enhance remyelination [10].

Betaine is a product of choline oxidation and occurs naturally in foods. Key food sources of betaine include cereal-derived foods and other plant foods such as spinach and beetroot [7]. Pasta, bread, spinach and breakfast cereals are commonly the main food sources contributing to betaine intake at the population level [11]. Betaine has antioxidant properties and may play a beneficial role in inflammation, obesity, diabetes, and in cardiovascular, liver and kidney health [12]. Betaine is a potent methyl donor, and has been shown to have neuroprotective effects in a study of experimental autoimmune encephalitis (EAE) mouse models [13].

While the findings of the aforementioned animal studies suggest a potentially beneficial role for supplemental choline and betaine in MS, the role of choline and betaine obtained from food sources in MS onset in humans is unclear. Hence, we tested associations between dietary choline and betaine intake and the likelihood of a first clinical diagnosis of central nervous system (CNS) demyelination (FCD) in participants of the Ausimmune Study.

## Methods

### Study population

The Ausimmune Study was a case–control study designed to collect data for investigating environmental risk factors for FCD [14]. It was conducted across four latitudinally different locations (Brisbane city 27° S, Newcastle region 33° S, Geelong and the Western districts of Victoria 37° S, and the island of Tasmania 43° S) in Australia with the goal of maximising recruitment of incident cases within each study region. Participants aged 18–59 years were recruited from 2003 to 2006 as reported elsewhere [14]. In brief, cases (n = 282) were notified to the study by clinicians after presenting with symptoms associated with CNS demyelination. The diagnoses were: a classic first demyelinating event (FDE), defined as a single, first, episode of clinical symptoms suggestive of CNS demyelination (n = 216); a first recognised event, but past history indicated a prior, undiagnosed event, that, on review was highly suggestive of CNS demyelination (n = 48); first presentation of primary progressive MS (based on neurologic assessment at recruitment) (n = 18). The specific diagnosis and date of onset were confirmed by an assessment with a neurologist. Control participants (n = 558) were recruited randomly through the Australian Electoral Roll, which has compulsory registration for citizens aged 18 + y. Up to four controls were matched by age (± 2 y), sex, and study region to each case.

### Ethical approval

Ethics approval for the Ausimmune Study was obtained from the Human Research Ethics Committees of the following participating institutions [14]: Barwon Health Research and Ethics Advisory Committee (ref. 03/46), Ballarat Health Services and St John of God Health Services Committee, Royal Brisbane and Women’s Hospital and Health Service District Office of the Human Research Ethics Committee (ref. 2003/093), The University of Queensland Medical Research Ethics Committee (ref. 2,003,000,253), The Princess Alexandra Hospital Human Research Ethics Committee (ref. 2004/059), The Queensland Institute of Medical Research Human Research Ethics Committee [ref. H0511-061 (P950)], Hunter Area Research Ethics Committee (ref. 03/06/11/3.07), Southern Tasmania Heath and Medical Research Ethics Committee (ref. H7436), The Australian National University Human Research Ethics Committee (ref. 2002/111). Participants provided written informed consent.

### Dietary assessment

We collected dietary intake data representative of foods consumed in the 12 months prior to the study interview. We used the Cancer Council Victoria Dietary Questionnaire for Epidemiological Studies version 2 (DQESv2), a self-administered, semiquantitative food frequency questionnaire [15]. The DQESv2 was designed to capture dietary intake in the ethnically diverse adult population in Australia. In a validation study that included 63 women of child-bearing age, the DQESv2 delivered mean intakes within ± 20% of 7-day weighed food records for 21 of 27 nutrients [16].

Choline and betaine composition data were obtained from a composition database for Australian foods [17] for the 101 DQESv2 food and beverage items, with the exception of potato crisps, bean sprouts and tofu. Food and beverage items from the composition database were grouped based on conceptual and nutritional similarities driven by choline. Average concentrations were calculated for each group of food and beverage items and matched to the food categories of the DQESv2. Data were obtained from the US FoodData Central (FDC) database for potato crisps (FDC ID 169677), bean sprouts (average concentration calculated from FDC IDs 169957, 168499, 169137, 168384) and tofu (average concentration calculated from FDC IDs 174290, 172476, 172451, 172449, 172448) [18].

In order to assess the association between choline and betaine intake and likelihood of FCD in the context of the overall diet, we used the alternate Mediterranean diet score (aMED) as a measure of diet quality [19]. The aMED was calculated from the DQESv2 dietary intake data using methods described previously [2, 19]. Briefly the aMED is an adaptation of the scale that was originally designed to score adherence to a traditional Mediterranean diet [20, 21]. For the aMED, consumption of each of the following components at or above the median intake (servings/day) score a value of 1: vegetables (except potato products); legumes; fruit; nuts; wholegrains. A value of 1 is also scored for: consumption of red and processed meat at less than median intake; fish at or above median intake; ratio of monounsaturated to saturated fat at or above the median intake; ethanol intake of 5–15 g/day (females), or 10–25 g/day (males) [2, 19]. A value of 0 is scored for each of the above criteria that are not met. Possible scores range from 0 to 9, with a higher score aligning with higher diet quality [19].

### Covariates

Participant characteristics were recorded by the person conducting the study interview (age, sex) or captured by self-administered questionnaires (study region [Brisbane, Newcastle, Geelong, Tasmania], education [Year 10 or below, Year 11 or 12, Technical and Further Education/Trade/Apprentice], smoking history [No/Yes], and history of infectious mononucleosis [No/Yes/Don’t know]). Height and weight were measured by the study nurse, and basal metabolic rate (BMR) was calculated using established equations [22]. Dietary misreporters were classified using a Goldberg cut-off below BMR × 1.05 [23] and defined as under-reporter or plausible/over-reporter. Participants provided a blood sample to measure serum 25-hydroxyvitamin D (25(OH)D) concentrations, which were deseasonalised [14].

### Statistical analyses and justification of sample size

The Ausimmune Study investigators aimed to recruit all incident cases within each of the study regions via referral from a range of clinicians, including neurologists, ophthalmologists and general physicians. Primary care physicians were also engaged by the study to capture any potential cases not identified through the aforementioned pathways. The response rate of notified cases was > 90%.

Participants who completed the DQESv2 and had plausible total energy intake (females, 500–3500 kcal/day; males, 800–4000 kcal/day) [24] (cases = 264, controls = 474) were included in statistical analyses. Missing data for covariates were tested for the probability of being missing [25]; participants with missing data were not excluded from analyses if the data were missing completely at random. Choline and betaine intakes and their combined total (choline + betaine) were investigated as continuous variables per 50 mg/day. Odds ratios (OR) with 95% confidence intervals (CIs) for associations between choline, betaine and choline + betaine intake and likelihood of FCD were calculated using logistic regression with full propensity score matching. Matching variables were selected because they: (1) were case–control study matching variables (sex, age, study region); (2) are well-established/potential risk factors for MS (education, smoking history, history of infectious mononucleosis, serum 25(OH)D concentration); and (3) reduce the risk of confounding by overall energy intake (total energy intake, dietary misreporting). We used two levels of covariate matching: in model 1, cases and controls were matched on the case–control study matching variables; in model 2, cases and controls were additionally matched on education, smoking history, history of infectious mononucleosis, serum 25(OH)D concentration, total energy intake and dietary misreporting.

To assess the association between choline and betaine intake and likelihood of FCD in the context of the overall diet, correlations and interactions between aMED and choline, betaine and choline + betaine were assessed. Weak correlations (correlation coefficients ~ 0.1) only were observed, with no significant interactions between aMED and choline, betaine and choline + betaine (*p* = 0.70, 0.08 and 0.90, respectively, for choline, betaine and choline + betaine). Therefore, we ran additional logistic regression models adjusting for aMED.

Final models were bootstrapped (500 replicates) for reporting of 95% CIs; non-bootstrapped models were used for reporting of ORs. The analyses were conducted on the total population and stratified by sex due to different patterns of incidence in MS over time in males and females, and previous studies showing differences in associations with dietary factors by sex. Statistical significance was set at *p* < 0.05. We used Stata/SE 18.0 [26].

## Results

Of the 282 cases and 558 control participants recruited to the Ausimmune Study, 264 and 474, respectively, had plausible energy intake and were included in the analyses. Missing data for education (1 case), history of smoking (1 case, 1 control), serum 25-hydroxyvitamin D concentration (7 cases, 24 controls), history of infectious mononucleosis (1 case), dietary misreporting (1 case, 3 controls) were missing completely at random. Participant characteristics are presented in Supplementary Table 1.

In line with the higher prevalence of MS in females than males, there were ~ 3.5 times more female participants than male participants. As expected, based on established risk factors for MS, a higher proportion of case participants had a history of smoking or a history of infectious mononucleosis compared to control participants, and the mean circulating concentration of 25(OH)D was lower in case participants than control participants. The median (interquartile range [IQR]) intake of choline was 336 (176) and 361 (170) mg/day for cases and controls, respectively. Respectively for cases and controls, the median (IQR) intake of betaine was 59 (39) and 63 (46) mg/day.

In fully adjusted models that included aMED as a measure of diet quality, higher dietary choline intake and combined choline + betaine intake were significantly associated with lower likelihood of FCD in females only (Table 1). There were no statistically significant associations in any other fully adjusted models for females. We found no statistically significant associations between choline or betaine intake and FCD in males.

**Table 1:** Associations between dietary intakes of choline and betaine and likelihood of a first clinical diagnosis of central nervous system demyelination in participants of the Ausimmune Study

Choline and betaine intake ^1^	Total population (264 cases, 474 controls)			Females (205 cases, 368 controls)			Males (59 cases, 106 controls)		
	OR	95% CI	*p*	OR	95% CI	*p*	OR	95% CI	*p*
*Choline intake (per 50 mg/day)*									
Multivariable model 1^2^	0.95	0.90, 1.02	0.14	0.92	0.84, 1.00	0.049	1.01	0.91, 1.13	0.79
Multivariable model 2^3^	0.95	0.89, 1.01	0.12	0.92	0.84, 1.00	0.047	0.99	0.88, 1.12	0.89
Multivariable model 3^4^	0.95	0.89, 1.02	0.14	0.91	0.84, 1.00	0.05	1.01	0.90, 1.13	0.86
*Betaine intake (per 50 mg/day)*									
Multivariable model 1^2^	0.87	0.72, 1.04	0.13	0.80	0.64, 1.02	0.07	0.95	0.66, 1.37	0.79
Multivariable model 2^3^	0.85	0.70, 1.02	0.08	0.79	0.61, 1,02	0.07	0.90	0.61, 1.33	0.59
Multivariable model 3^4^	0.85	0.70, 1.03	0.10	0.79	0.61, 1.02	0.07	0.97	0.66, 1.42	0.87
*Combined choline + betaine intake (per 50 mg/day)*									
Multivariable model 1^2^	0.96	0.91, 1.01	0.11	0.93	0.87, 0.99	0.03	1.01	0.92, 1.10	0.89
Multivariable model 2^3^	0.96	0.91, 1.01	0.08	0.92	0.86, 0.99	0.03	0.99	0.90, 1.09	0.79
Multivariable model 3^4^	0.96	0.91, 1.01	0.10	0.92	0.86, 0.99	0.03	1.01	0.91, 1.11	0.92

*CI* confidence interval, *IQR* interquartile range, *OR* odds ratio

^1^Choline intake (mg/day), median (IQR): total population: cases, 336.5 (176.0); controls, 361.0 (170.0); females: cases, 319.0 (125); controls, 340.6 (151.0); males: cases: 466.1 (205.0), controls: 461.7 (220.0); Betaine intake (mg/day), median (IQR): total population: cases: 58.4 (39.0), controls: 63.1 (46.0); females: cases: 53.5 (32.0), controls: 57.5 (36.0); males: cases: 91.0 (65.0); controls, 89.5 (69.0); Combined choline + betaine intake (mg/day), median (IQR): total population: cases: 401.1 (203.0), controls: 424.4 (210.0); females: cases: 377.6 (142.0), controls: 402.2 (181.0); males: cases: 555.2 (246.0), controls: 553.3 (271.0)

^2^Model 1: logistic regression with full propensity matching (age, sex, study region), bootstrapped with 500 replicates. Sex was removed from matching when the analysis was stratified by sex

^3^Model 2: logistic regression with full propensity matching (age, sex, study region, education, history of smoking, history of infectious mononucleosis, deseasonalised serum 25-hydroxyvitamin D concentration, total energy intake, and dietary misreporting), bootstrapped with 500 replicates. Sex was removed from matching when the analysis was stratified by sex

^4^Model 3: logistic regression with full propensity matching (age, sex, study region, education, history of smoking, history of infectious mononucleosis, deseasonalised serum 25-hydroxyvitamin D concentration, total energy intake, dietary misreporting and the alternate Mediterranean diet score (aMED)), bootstrapped with 500 replicates. Sex was removed from matching when the analysis was stratified by sex

## Discussion

To our knowledge, this is the first published study to investigate dietary choline and betaine intakes in relation to FCD or MS onset in humans. Independent of diet quality, higher choline intake and combined choline and betaine intake were associated with lower likelihood of FCD in females only.

Our finding that higher choline intake associates with lower likelihood of FCD complements the findings from an animal model of MS showing that an intermediate molecule in the choline metabolism pathway, cytidine-5′-diphosphocholine, promotes regeneration of demyelinated neurons [10]. However, while we found an association between choline and choline + betaine intake and lower likelihood of FCD in females only, that animal model study included only male animal models [10]. The reason for the difference by sex in our study is unclear. It could be attributed to low statistical power for males, with the possibility that no true difference exists by sex. The smaller sample of males compared to females in our study reflects the disproportionate prevalence of MS by sex, with the estimated global prevalence of MS being at least double among females (69%) compared to males (31%) [27].

If any difference in association between choline/choline + betaine intake and likelihood of FCD exists by sex, it could be due to differences in dietary intake and/or pathological processes by sex. Our results also support our previous findings from the Ausimmune Study that consumption of choline-rich foods, such as unprocessed red meat and fish, were associated with lower likelihood of FCD in the total population and females, respectively [3, 28]. Healthier dietary patterns that are characterised by higher consumption of choline-containing lean protein and betaine-containing plant foods were associated with lower likelihood of FCD in the total population [2, 4]. However, meat, fish and plant/cereal foods are also sources of other nutrients (e.g., omega-3 fatty acids, B vitamins and vitamin D) that may be beneficial with respect to MS onset [3, 28, 29]. Male participants of the Ausimmune Study consumed more red meat [3] and fish than female participants. Differences by sex were also observed in our team’s prior study of a healthy dietary pattern, which is characterised by higher consumption of poultry, fish, eggs, vegetables and legumes [4]. In that study, a one standard deviation increase in the healthy dietary pattern score was associated with a 28% lower likelihood of FCD (adjusted OR [aOR] 0.72; 95% CI 0.56, 0.93) in female participants of the Ausimmune Study [4]. In male participants the same association was non-significant (aOR 0.91; 95% CI 0.43, 1.93) [4], albeit in the same direction and with the same low statistical power limitation for males as in the present study. Further research is needed to determine if any difference in association between diet and likelihood of FCD exists by sex and, if so, whether it is driven by specific foods or nutrients, or any sex-specific (diet-related or unrelated) pathological processes.

There are plausible mechanisms for a role of choline and betaine in the pathogenesis of MS. Although there has been limited research to date, initial studies have suggested that both choline and betaine may influence myelination, with demyelination being the hallmark of MS. In MS, chronic inflammation of the central nervous system leads to demyelination, neurodegeneration and axonal loss in the white and grey matter of the brain and spinal cord [30]. Choline is a primary dietary source of methyl groups, and a precursor to the neurotransmitter acetylcholine and components of cell membranes, especially myelin [31]. Through its oxidation to form betaine, a methyl group donor that supports regeneration of methionine, choline plays a key role in the one-carbon cycle [32]. Methionine synthesis and the one-carbon cycle are key to CNS function [33]. Betaine can be metabolised endogenously from choline, or obtained directly from the diet. Betaine has been shown to modulate the immune system and lessen extensive demyelination in animal models of MS [13]. The authors of that study suggested a potential mechanism by which betaine inhibits secretion of IL-6 by dendritic cells, impairing the differentiation of T cells that secrete IL-17 [13]. T cells that secrete IL-17 have been implicated in the pathogenesis of MS [34]. Although not reaching statistical significance, the association between betaine and likelihood of FCD was in the expected direction (coefficients < 1.00 and *p-*values < 0.10). It is important to note that dietary intake of betaine does not account for endogenous synthesis. While dietary betaine is thought to be highly and rapidly absorbed and distributed [35], genetic variation in, and dietary impact on, microbiota composition and its influence on choline bioavailability requires further research [36, 37]. Yet, it is conceivable that dietary choline and betaine could influence MS onset via myelination pathways.

The potential mechanisms underlying our hypothesis that choline/betaine intake could be associated with likelihood of FCD are based on studies of animal models. Animal models are acknowledged as the most useful available resource for exploring potential mechanisms in MS onset; toxin-induced models, such as the toxic cuprizone-induced mouse model are considered ideal for studies of the remyelination process, while the EAE model well-represents autoimmune pathogenesis [38]. However, they are also subject to limitations such as not representing the wide variation in presentation of MS in humans, stark differences in the timing of symptom onset in mice compared to humans, lack of genetic heterogeneity and inbred genetic irregularities in purpose-bred animal models that may not reflect genetic characteristics across human populations, and between-species differences in immune systems [38].

Furthermore, the role of diet in MS is emerging and there is much that is not understood about associations between foods and their components and MS onset, and the underlying mechanisms. Nevertheless, in a new and understudied area of research, the aforementioned animal studies and our present study provide important foundational insight into food components that may influence MS, and suggest which other components and foods may correspond to any relationships. Given that there is no known cure for MS and other known risk factors (e.g., history of infectious mononucleosis, latitude of residence) may be beyond the control of the individual, the feasibly modifiable nature of diet makes it an important risk factor to understand. Thus, findings such as those from our present study are important in informing and directing much-needed ongoing research into the role of diet in MS – both its onset and progression.

A strength of our study was the use of data from the Ausimmune Study, a large, multi-centre, matched case–control study. It was specifically designed to capture known environmental risk factors for MS, such as vitamin D status, history of smoking and geographical latitude, which were accounted for in our analysis. However, historical anthropometric data were not collected to allow consideration of adolescent obesity, which has emerged as a risk factor for MS [39, 40] since the design of the Ausimmune Study [14].

A limitation of the study was that the DQESv2 food frequency questionnaire did not capture consumption of liver, one of the richest food sources of choline [18]. However, liver was not reported as widely consumed during the National Nutrition and Physical Activity Survey that was conducted in Australia in 2011–2012 [41]; of 12,153 participants who completed a 24-h dietary recall on day 1, only 36 reported consuming liver. Self-reported food consumption data are subject to widely acknowledged limitations such as recall bias and measurement error; however, we included only participants with plausible total energy intake and included dietary a misreporting variable in Model 2. The Ausimmune Study captured dietary intake data representative of the 12 months preceding the study interview and at the time of FCD; however, the pathogenesis of MS may occur over a longer period than 12 months. Dietary intake > 12 months prior to FCD was not captured. The observational nature of the Ausimmune Study precludes the ability to establish causation. A further limitation was that the study population was primarily Caucasian, limiting generalisability to other populations, and the participation rate was lower for controls than cases [42]. Further, our study relates to choline and betaine intake and the likelihood of MS onset, which is important for those at high risk of MS. Our study did not investigate any role of choline or betaine intake in disease progression outcomes in people who already have MS. This merits separate research, as studies of associations between diet and MS progression require different methodological considerations to those investigating disease onset.

## Conclusion

Higher choline and choline + betaine intake were associated, independent of diet quality as measured by a Mediterranean diet score, with lower likelihood of FCD in females. Given the potential beneficial role of choline and betaine, testing associations with MS onset in other populations globally is warranted to support our findings.

## Supplementary Information

Below is the link to the electronic supplementary material.

Supplementary file1 (DOCX 15 KB)

## Data Availability

The data analysed in this study were obtained from the Ausimmune Study. Requests to access the data via a collaborative agreement should be directed to Professor Anne-Louise Ponsonby, annelouise.ponsonby@florey.edu.au.
