# Supplementary material for: Higher dietary choline and betaine intakes are associated with lower likelihood of central nervous system demyelination in Australian women
Source: Eur J Nutr. 2026 Feb 14;65(2):56. doi: 10.1007/s00394-026-03915-x (PMC12906510; doi:10.1007/s00394-026-03915-x)
Supplement: Supplementary file 1 — Supplementary file1 (DOCX 15 KB) [file 394_2026_3915_MOESM1_ESM.docx]

**Supplementary Table 1** Participant characteristics

| **Characteristics** | **Controls (n=474)** | **Cases**  **(n=264)** |
| --- | --- | --- |
| **Covariates** | | |
| Age (years), median (IQR) | 39.8 (14.7) | 38.8 (14.7) |
| Sex |  |  |
| Males | 106 (22.4) | 59 (22.3) |
| Females | 368 (77.6) | 205 (77.7) |
| Study region, n (%) |  |  |
| Brisbane (latitude 27^◦^S) | 165 (34.8) | 90 (34.1) |
| Newcastle (latitude 33^◦^S) | 77 (16.2) | 34 (12.9) |
| Geelong (latitude 37^◦^S) | 125 (26.4) | 64 (24.2) |
| Tasmania (latitude 43^◦^S) | 107 (22.6) | 76 (28.8) |
| Education, n (%) |  |  |
| Year 10 or below | 157 (33.1) | 65 (24.7) |
| Year 11 or 12 | 69 (14.6) | 52 (19.8) |
| TAFE/Trade/Apprentice | 125 (26.4) | 79 (30.0) |
| University | 123 (25.9) | 67 (26.5) |
| History of smoking, n (%) |  |  |
| No | 227 (48.0) | 103 (39.2) |
| Yes | 246 (52.0) | 160 (60.8) |
| History of infectious mononucleosis, n (%) |  |  |
| No | 384 (81.0) | 172 (65.4) |
| Yes | 71 (15.0) | 73 (27.8) |
| Don’t know | 19 (4.0) | 18 (6.8) |
| 25(OH)D (nmol/L), mean (SD) | 81.7 (30.3) | 76.1 (29.8) |
| Total energy intake (kcal/day), median (IQR) | 1723.3 (849.0) | 1657.8 (855.0) |
| Dietary misreporting, n (%) |  |  |
| Under-reporter | 107 (22.7) | 73 (22.8) |
| Plausible/over-reporter | 364 (77.3) | 190 (77.2) |
| Choline intake (mg/day), median (IQR) | 361.0 (170.0) | 336.5 (176.0) |
| Betaine intake (mg/day), median (IQR) | 63.1 (46.0) | 59.4 (39.0) |
| aMED, median (IQR) | 4 (3) | 4 (2) |
| **Other characteristics** | | |
| Physical activity^1^ |  |  |
| High | 169 (36.4) | 105 (41.2) |
| Moderate | 176 (37.9) | 92 (36.1) |
| Low | 119 (25.7) | 58 (22.8) |
| Body mass index, median (IQR) | 25.8 (7.7) | 25.9 (7.6) |
| Proportion of energy from fat, median (IQR) | 35.7 (8.5) | 36.3 (6.5) |
| Proportion of energy from protein, median (IQR) | 18.9 (3.6) | 18.5 (4.1) |
| Proportion of energy from carbohydrate, median (IQR) | 41.2 (8.3) | 42.4 (8.8) |
| Consumption of foods and beverages (g/day), median (IQR) |  |  |
| Fruit | 214.7 (217.8) | 220.4 (208.5) |
| Vegetables | 123.4 (76.4) | 117.4 (72.1) |
| Cereals/grains | 183.5 (127.8) | 170.3 (108.1) |
| Legumes | 4.6 (9.3) | 3.7 (9.5) |
| Nuts | 2.5 (5.2) | 2.3 (6.1) |
| Dairy & dairy alternatives | 265.7 (186.5) | 376.8 (187.0) |
| Red meat, including processed meat | 89.5 (78.4) | 80.2 (82.1) |
| Chicken | 25.6 (27.8) | 25.6 (29.3) |
| Fish, including fried fish | 23.8 (29.0) | 20.8 (28.6) |
| Eggs | 12.9 (6.0) | 12.9 (6.0) |
| Mixed dishes | 34.3 (41) | 36.2 (37.9) |
| Butter and margarine | 14.0 (17.5) | 14.0 (17.5) |
| Snacks/others | 70.7 (64.9) | 67.4 (66.6) |
| Tea & coffee | 750.0 (750.0) | 625.0 (821.4) |
| Alcoholic drinks | 60.5 (209.7) | 38.4 (172.4) |

25(OH)D, 25-hydroxyvitamin D; aMED, alternate Mediterranean diet score; IQR, interquartile range; SD, standard deviation; TAFE: Technical and Further Education.

The following had missing data: education (1 case); history of smoking (1 case, 1 control); serum 25-hydroxyvitamin D concentration (7 cases, 24 controls); history of infectious mononucleosis (1 case); dietary misreporting (1 case, 3 controls); body mass index (3 cases, 1 control).

^1^Data collected using the short form of the International Physical Activity Questionnaire (Craig CL, Marshall AL, Sjöström M, Bauman AE, Booth ML, Ainsworth BE. Pratt M, Ekelund U, Yngve A, Sallis JF, Oja P (2003) Med Sci Sports Exerc 35(8):1381-1395 https://10.1249/01.MSS.0000078924.61453.FB
